# Hybrid membrane distillation reverse electrodialysis configuration for water and energy recovery from human urine: An opportunity for off-grid decentralised sanitation

**DOI:** 10.1016/j.memsci.2019.05.010

**Published:** 2019-08-15

**Authors:** E. Mercer, C.J. Davey, D. Azzini, A.L. Eusebi, R. Tierney, L. Williams, Y. Jiang, A. Parker, A. Kolios, S. Tyrrel, E. Cartmell, M. Pidou, E.J. McAdam

**Affiliations:** a School of Water, Energy and Environment, Cranfield University, Bedfordshire, MK43 0AL, UK; b Department of Materials, Environmental Sciences and Urban Planning, Università Politecnica delle Marche, Piazza Roma, Ancona, Italy; c Naval Architecture, Ocean and Marine Engineering, University of Strathclyde, Glasgow, UK; d Scottish Water, Castle House, Carnegie Campus, Dunfermline, UK

**Keywords:** Reverse electrodialysis (RED), Recycle, Closed-loop, Salinity gradient energy

## Abstract

The integration of membrane distillation with reverse electrodialysis has been investigated as a sustainable sanitation solution to provide clean water and electrical power from urine and waste heat. Reverse electrodialysis was integrated to provide the partial remixing of the concentrate (urine) and diluate (permeate) produced from the membrane distillation of urine. Broadly comparable power densities to those of a model salt solution (sodium chloride) were determined during evaluation of the individual and combined contribution of the various monovalent and multivalent inorganic and organic salt constituents in urine. Power densities were improved through raising feed-side temperature and increasing concentration in the concentrate, without observation of limiting behaviour imposed by non-ideal salt and water transport. A further unique contribution of this application is the limited volume of salt concentrate available, which demanded brine recycling to maximise energy recovery analogous to a battery, operating in a ‘state of charge’. During recycle, around 47% of the Gibbs free energy was recoverable with up to 80% of the energy extractable before the concentration difference between the two solutions was halfway towards equilibrium which implies that energy recovery can be optimised with limited effect on permeate quality. This study has provided the first successful demonstration of an integrated MD-RED system for energy recovery from a limited resource, and evidences that the recovered power is sufficient to operate a range of low current fluid pumping technologies that could help deliver off-grid sanitation and clean water recovery at single household scale.

## Highlights

• Membrane distillation with reverse electrodialysis allows off-grid sanitation.
• Heat and concentrate development with MD enhance RED power density.
• The complex salt matrix within the feed does not limit power density.
• Good energy recovery from small salt concentrate volumes with recycle.

## Graphical abstract

**Figure:**
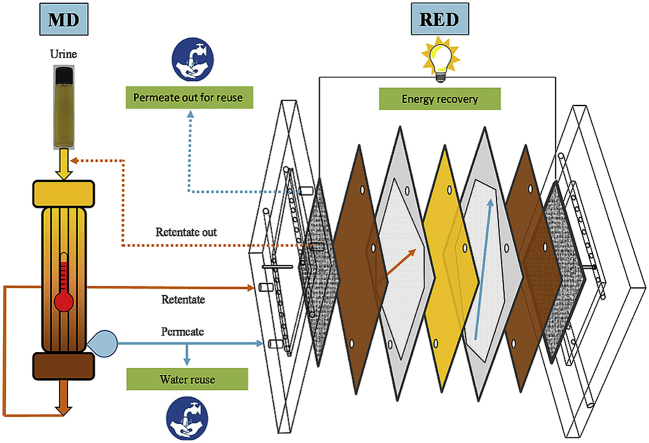

## Nomenclature and abbreviations

**List of symbols**
*A*: Cross sectional area of one membrane (m^2^)
C: Concentration (mol L^−1^)
Δ_mix_G: Gibbs free energy of mixing (J)
*ED*: Energy density (J kg^−1^)
*F*: Faraday constant (96485.33C mol^−1^)
*G*: Gibbs free energy
*H*: Enthalpy
*I*: Current (A)
*I*_*d*_: Current Density (A m^−2^)
*J*_*s*_: Total salt flux (mol m^−2^ s^−1^)
*J*_w_: Total water flux (mol m^−2^ s^−1^)
κ: Solution conductivity
*L*_*p*_: Average water permeability coefficient for both anion and cation exchange membranes (kg m^−2^ s^−1^ kg^−1^)
*M*: Molar mass of water (kg mol^−1^)
*m*: Mass (kg)
*N*: Number of cell pairs in membrane stack
*n*: Moles (mol)
*P*: Power (W)
*P*_*d*_: Power density (W m^−2^)
*P*_*s*_: Average salt permeability coefficient for the anion and cation exchange membrane pair (m^2^ s^−1^)
R: Universal gas constant (8.314 J K^−1^ mol^−1^)
*S*: Molar entropy (J K^−1^ mol^−1^)
*T*: Temperature (K)
t_w_: Number of water molecules transported with salt ions across the membranes (mol_water_ mol _salt_^−1^)
*U*: Potential (V)
*v*: Number of moles in 1 mol of salt
*z*: Valency of the ion
*α*: Permselectivity (%)
*Δμ*_*s*_: Difference in chemical potential of salt
*Δμ*_*w*_: Difference in chemical potential of water
*δ*_*m*_: Average membrane thickness of the anion and cation exchange membrane pair (m)
*γ*: Activity coefficient
∅: Osmotic coefficient (unitless)
*η*: Energy extraction efficiency (%); Subscripts; *B* Mixed concentrate and diluate; *C* Concentrate
*D*: Diluate
*G*: Gibbs (e.g. *P*_*G*_ = Gibbs power)
*Stack*: Measure across the membrane stack
*s*: Salt (e.g. Js = total salt flux)
*w*: Water; Abbreviations; SDG Sustainable development goals
MD: Membrane distillation
RED: Reverse electrodialysis; NOM Natural organic matter; VMD Vacuum membrane distillation; COD Chemical oxygen demand; NH_4_^+^-N Ammonical nitrogen; NaCl Sodium chloride; OCV Open circuit voltage
LC: Low concentration (refers to channel)
HC: High concentration (refers to channel)
SOC: State of charge

## 1 Introduction

Sustainable small scale sanitation systems treating blackwater on-site have been recently innovated to address the water sustainable development goals (SDG 6) in low income countries [1,2]. Source separation is an accepted practice in Europe [6], and is advantageous in decentralised innovations, since upstream solids/liquid separation [7,8] advantages technology selection and energy demand for downstream processing [[3], [4], [5]]. Improving regulatory practice in low income countries (LICs) now means new technologies are required to meet international discharge standards for water reuse or discharge [9]. Membrane technology is deemed a practicable choice for liquid phase treatment, providing a definitive barrier to pathogens, within a modular and comparatively small footprint [[10], [11], [12]]. However, in many cases, electricity supplies are unsafe and unreliable [13]. In contrast, sources of waste heat are comparatively abundant in LICs, for example through solar or domestic activities (wood burning stoves) [14]. Consequently, thermally driven membrane separation offers significant opportunity in LICs for post source separation treatment of the liquid phase, which comprises primarily of urine; and has been successfully demonstrated with membrane distillation (MD) for water recovery in various space missions [10]. A further source of waste heat is in the direct combustion of human faeces (solid phase) which releases sufficient thermal energy to introduce the necessary vapour pressure gradient for thermal membrane separation, since its calorific value is equivalent to brown coal [15,16].

Whilst MD primarily requires heat, some electrical energy is inevitably demanded which necessitates the identification of an alternative energy source to that of distributed networks, which lack penetration and are often unreliable [13]. Membrane distillation produces two outputs from urine treatment: a high quality permeate with an incredibly low concentration of inorganic ions (∼0.2 mS cm^−1^); and, a salt rich retentate exceeding 20 mS cm^−1^ (Table 2). On the assumption of the selective remixing of these two solutions, the release of Gibbs free energy exceeding 337 J kg^−1^ can be realised; this further permits partial management of the retentate, which could increase the concentration factor (or product conversion) that can be achieved with MD whilst only increasing permeate conductivity by a small amount. The Gibbs free energy available can be harnessed as electrical energy via reverse electrodialysis (RED) which uses an alternating series of cation and anion exchange membranes separating concentrated and dilute solutions to produce a salinity gradient. The selective flow of anions and cations through the respective membranes creates an electrochemical potential across the stack, where at the electrodes, a redox reaction converts the ionic flow to an electric current. Reverse electrodialysis has gained considerable interest since the first demonstration by Pattle in 1954 where a gross power density of 0.05 W m^−2^ was reported [18,19]. Since then, research has been predominantly directed towards sodium chloride based salinity gradients (seawater and concentrated brines) and thermolytic salts, as recently reviewed by Mei and Tang [20], and Tufa et al. [21] Research advances in these areas have focused on maximising power density through the optimisation of module design, membrane materials, fouling mitigation and operational conditions [20,21]. As a result, higher power densities of 2.2 W m^−2^ for seawater/river water applications (at ambient temperature) have been realised using modified membranes [22], with theoretical values predicted at 4.2 W m^−2^ [23], demonstrating the progress and potential of RED with optimisation.

Whilst salinity gradient technologies can be applied to a broad range of environmental matrices, few studies have approached RED for less conventional saline wastewaters, which could provide wider opportunities for energy recovery and discharge management. Kingsbury et al. challenged a RED stack with multiple real waters including municipal wastewater effluent, and pickling brine as dilute and concentrate examples. It was concluded that organic matter within the dilute stream was the main hindrance to power density (up to 43%), with inorganic solutes or organics in the concentrate presenting little effect [24,25]. However, the application also determines how best to optimise energy recovery from RED. For example, in several hybrid RED applications, reverse osmosis [26,27], electrodialysis [28] and solar evaporation [27], have been used to further concentrate salinity gradients for higher power densities and discharge management, which draws close parallels to the complementary proposed with MD [29]. The critical distinction between these previous studies, and that of RED as an ‘off grid’ solution for energy recovery from decentralised sanitation systems is that the solution volume will be finite. In such a resource constrained environment, the challenge is therefore in maximising energy recovery from the available volume of saline solution, which expectedly necessitates a recycle to maximise energy recovered per unit of salt, as this becomes the critical focus, rather than the maximisation of power density with single-pass flow, which is generally prioritised in larger-scale applications for which both solutions are in abundance.

Importantly, the scalability of RED has been demonstrated from feed water flows of 2.34 ton h^−1^, 250 m^2^ surface area and power production of 95.8 W [30], to microfluidic and nano-scale devices [31,32] which evidences the potential to scale-down to the size of an ‘off-grid’ decentralised sanitation system. The synergistic partnership between MD and RED at this scale would demand limited capital cost, with the potential to enable dependable local sanitation and the provision of high quality water (MD facilitated by waste heat), whilst providing a complementary source of stable power to support treatment (RED facilitated by MD salinity gradient) in an environment where such services and products are economically inaccessible for many [2,13]. This study therefore aims to evaluate the synergistic potential of a MD-RED configuration for small scale decentralised sanitation systems to enable electrical energy recovery in co-operation with the provision of safe sanitation from thermally driven membrane technology. Specific objectives are to: (i) understand the impact of the urine salt matrix on energy recovery through decoupling urine constituents into discrete groups; (ii) establish operational boundary conditions (feed concentrations, temperature, flowrate) using single-pass feed fluid flow for characterisation of peak power density; (iii) determine energy extraction efficiency and recovery with feed fluid flow in recycle mode by comparing experimentally obtained energy to the theoretical Gibbs free energy in recycle mode; and (iv) demonstrate MD-RED using real urine (concentrate) and MD permeate (diluate) recovered from urine treatment.

## 2 Materials and methods

### 2.1 Chemicals and solutions

All chemicals required for the preparation of synthetic urine and electrode rinse solution were sourced from Fisher Scientific (Loughborough, UK) or Sigma Aldrich (Dorset, UK) as laboratory grade. Deionised water was taken from a PURELAB Elga system (18 ΩM-cm at 25 °C). The composition of the synthetic urine was adapted from analysis by Putnam which detailed several specific groups of constituents: inorganic salts, organic ammonium salts, and organic compounds, providing a total ionic concentration of 248 mEq L^−1^ (Table 1) [17]. The synthetic urine was benchmarked against several fluids of equivalent charge to aid diagnosis of governing separation phenomena, including a sodium chloride (NaCl) control (248 mEq L^−1^ as NaCl) and an inorganic control comprised of monovalent and divalent salts, representative of those present in human urine (248 mEq L^−1^). Real human urine was collected by consenting anonymous volunteers through a regime approved by Cranfield University's Research Ethics System (Project ID 2384), and used directly without dilution or pre-treatment. Storage of real urine was at 4 °C and used or discarded within three days of collection.

**Table 1:** Summary of synthetic urine recipe adapted from Putnam (1971).

Urine category	Chemical group	Compound	Typical concentration (mg L^−1^)	Molar concentration (Mol L^−1^)	Cations	Anions	mEq L^−1^
Control	Sodium chloride	Sodium chloride	14497	0.248	Na^+^	Cl^−^	248.062
Inorganic salts	Monovalent	Sodium chloride	8001	0.137	Na^+^	Cl^−^	136.91
		Potassium chloride	1641	0.022	K^+^	Cl^−^	22.012
		Potassium bicarbonate	661	0.0066	K^+^	HCO_3_^−^	6.6
	Multivalent	Potassium sulphate	2632	0.0151	K^+^	SO_4_^−2^	30.2
		Magnesium sulphate	783	0.0065	Mg^2+^	SO_4_^−2^	13.01
Synthetic urine	Inorganic salts	Sodium chloride	9524	0.137	Na^+^	Cl^−^	162.98
		Potassium chloride	1951	0.022	K^+^	Cl^−^	26.2
		Potassium bicarbonate	790.79	0.0066	K^+^	HCO_3_^−^	7.9
		Potassium sulphate	3133	0.0151	K^+^	SO_4_^−2^	35.95
		Magnesium sulphate	932.83	0.0065	Mg^2+^	SO_4_^−2^	15.5
	Organic ammonium salts	Ammonium hippurate	1250	0.0064	NH_4_^+^	C_6_H_5_CO·NHCH_2·_CO_2_^−^	6.4
		Ammonium formate	88	0.0014	NH_4_^+^	HCO_2_^−^	1.4
		Ammonium citrate	756	0.0034	NH_4_^+^	HC_6_H_5_O_7_^−2^	6.8
		Ammonium lactate	394	0.0037	NH_4_^+^	C_3_H_5_O_3_^−2^	7.4
	Organic compounds	Urea	13400	0.22			
		Creatinine	1504	0.0132			
		Creatine	373	0.0026			
		Glycine	315	0.0042			

### 2.2 Reverse electrodialysis cell

**Figure 1:**
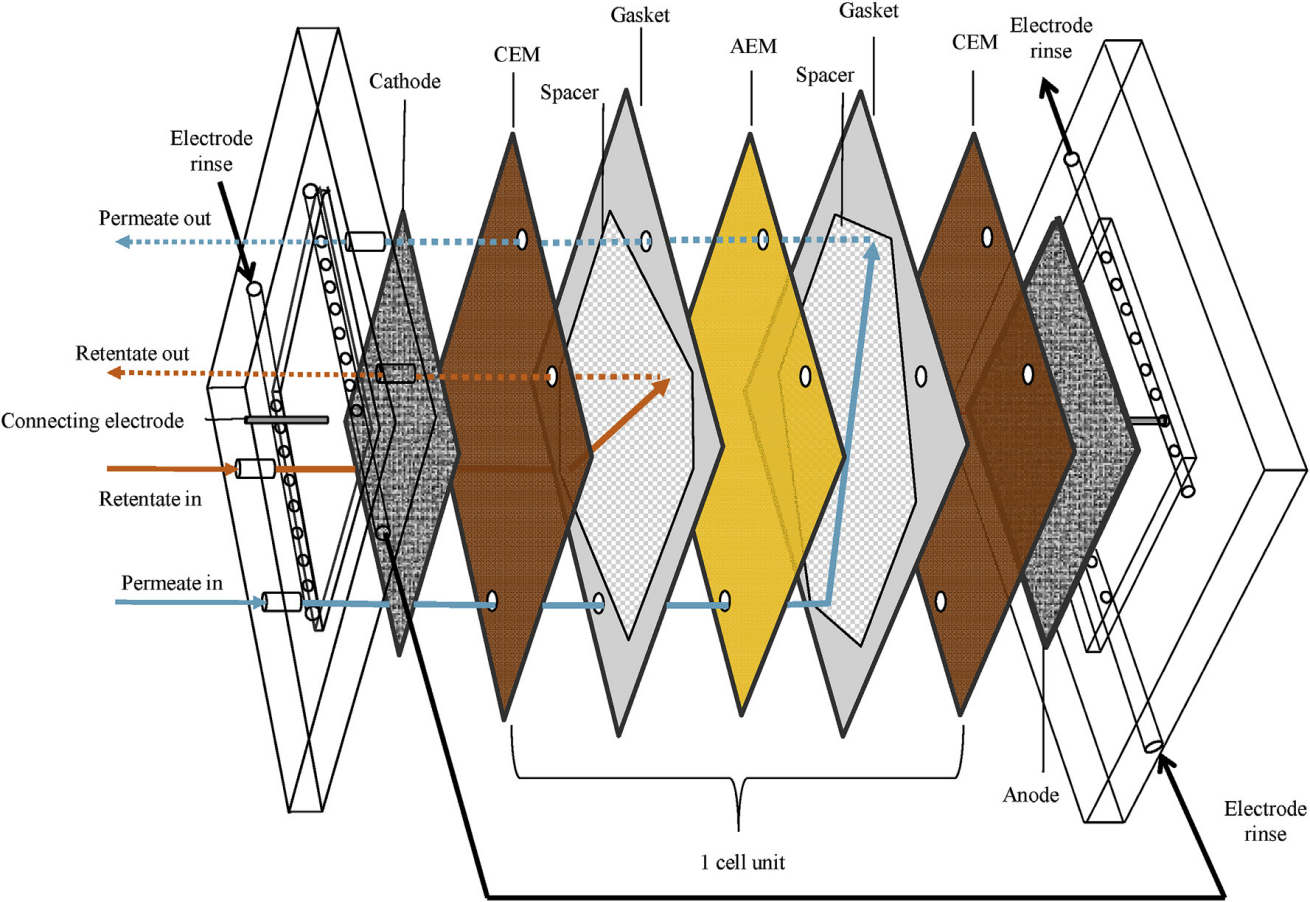
Schematic of reverse electrodialysis cell used in this study.

The custom RED stack used throughout this study is illustrated in Fig. 1. The endplates were fabricated from acrylic (Model Products, Bedford, UK) with stainless steel bolts to secure the stack. The membrane stack consisted of 5 repeating cell units of anion and cation exchange membranes (Neosepta AMX and CMX, Eurodia, France) with an effective area of 100 cm^2^ per membrane. These were sealed with silicon gaskets (Silex Silicones, UK) and nylon spacers with an open area of 35% (Sefar, UK) both 0.3 mm in thickness. The concentrate and diluate were pumped through the stack in a co-current configuration with peristaltic pumps. Titanium mesh plate electrodes coated with a Ru/Ir mixed metal oxide (MMO) (10 cm × 10 cm, Magneto, Netherlands) were fixed within the endplates of the stacks and acted as anode and cathode. An electrode rinse solution of 0.25 M NaCl was continuously circulated within the electrode compartments at a flow rate of 100 mL min^−1^ using a peristaltic pump (Watson Marlow, UK). Galvanostatic measurements were conducted using an Iviumstat. h (Alvatek, UK). Current was applied across the mesh working electrodes and Ag/AgCl reference electrodes (QM711X, ProSense BV) were placed in the anolyte/catholyte to measure electrical potential across the RED stack.

**Fig. 2:**
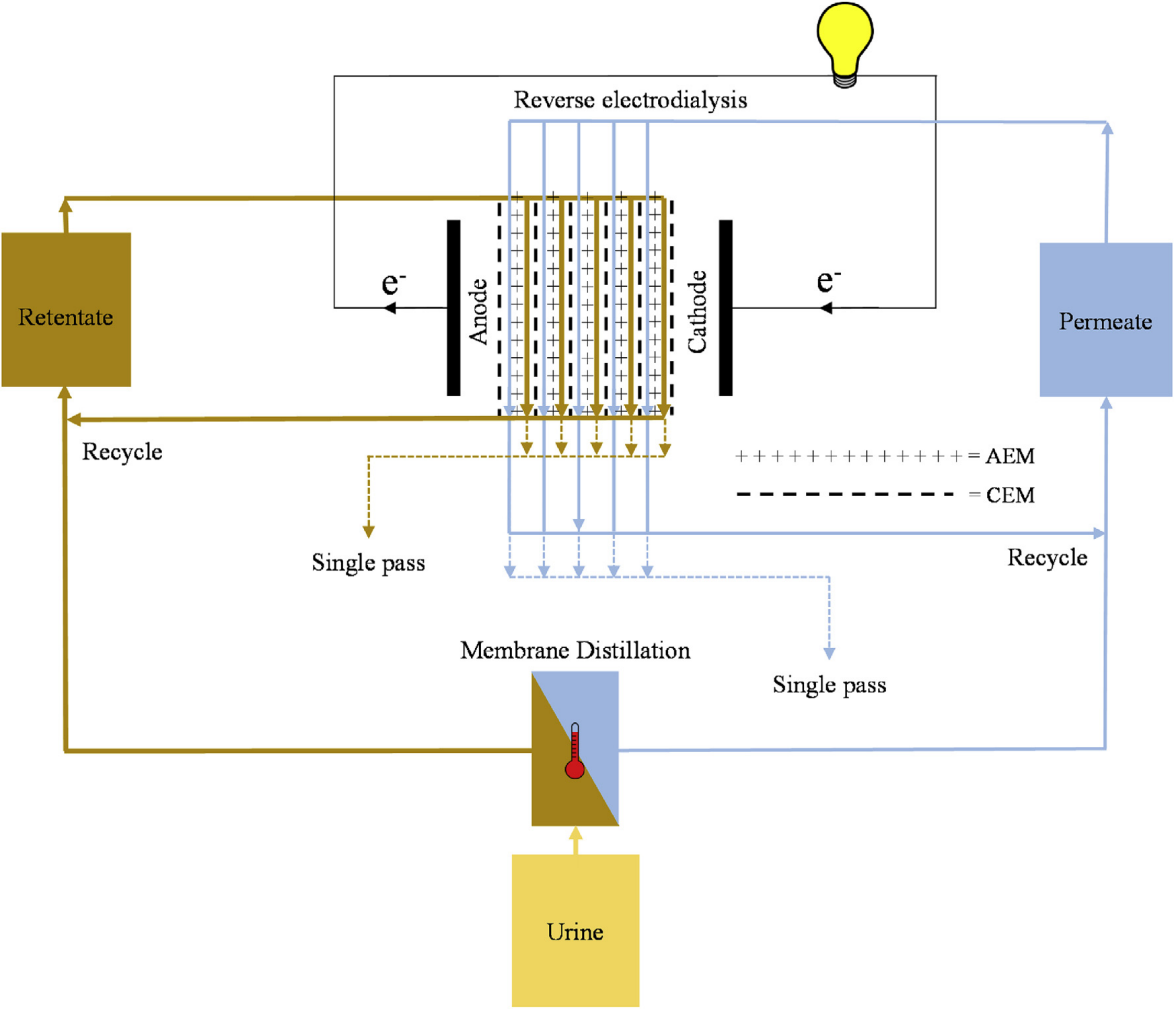
Schematic of the operation modes (single pass, recycle) practiced in this study.

To determine the ability of RED to convert the Gibbs free energy of urine to electrical power and directly compare with previous studies of RED using traditional electrolytic solutions (i.e. sea water/river water), the system was initially tested in a single-pass configuration (typically used to determine maximum power density when the electromotive force is at its greatest potential). In this arrangement, the solutions passed directly through the stack and the influent concentrations of the diluate and concentrate were therefore constant and the solutions exiting the stack discarded (Fig. 2). Galvanostatic polarisation measurements were conducted and the current was scanned at a rate of 0.2 mA s^−1^ from 0 to the maximum value, when the voltage of the stack reversed [33].

The available volume of urine in any system will ultimately be finite. To utilise the full Gibbs free energy stored within the MD retentate, a recycle configuration was utilised to enable the complete mixing of the retentate and permeate within the RED stack (Fig. 2). Consequently, the system was discharged at a constant current to mimic analogous discharge studies of galvanostatic cycling tests conducted on batteries and concentration gradient flow batteries [[34], [35], [36], [37]]. Constant current discharge experiments were conducted where 1 L of concentrate and diluate were recirculated through the stack until the potential across the stack reversed. This allowed for determination of the extractable energy efficiency and energy recovery of the RED stack. The conductivity of the bulk concentrate and diluate was recorded with conductivity probes (CDH SD1, Omega, UK). To measure water flux through the membranes, the concentrate and diluate were each placed on balances (Symmetry PT-4202E, Cole Parmer, UK) for the duration of the experiment. However, no significant change in mass was observed during the course of the experiments. This is likely due to the small osmotic pressure difference between the diluate and concentrate (≤9.9 bar), low water permeance (∼0.002 L m^−2^ h^−1^ bar^−1^) of the ion exchange membranes [38] and the relatively short time scales of the experiments (<24 h).

### 2.3 Membrane distillation

Vacuum membrane distillation (VMD) was used to recover high quality water from real urine, whilst also producing a urine concentrate, rich in inorganic salts, as the retentate (Fig. S1). The feed was heated in a water bath (TC120, Grant, UK) at 40 °C whilst being recirculated through the lumen of the membrane module (G542, MiniModule, Membrana, DE) using a peristaltic pump (520S, Watson Marlow, UK). A vacuum was applied to the shell side of the membrane and the permeate condensed at 2 °C with a glass condenser connected to a heater chiller (GD120, Grant, UK). The concentrated urine feed and permeate were stored at < 5 °C until use. The characteristics of the MD feed, permeate and retentate expressed as chemical oxygen demand (COD), ammoniacal nitrogen (NH_4_^+^-N) and conductivity is presented in Table 2.

**Table 2:** Real urine characteristics and membrane distillation permeate trialled in this study.

	Conductivity (mS cm^−1^)	pH	NH_4_^+^-N (mg L^−1^)	COD (mg L^−1^)
Urine 1 x Concentrated	12.65	6.585	207	6330
Urine 2 x Concentrated	24.8	6.92	548	9630
Permeate	0.207	8.5	38.9	253

## 3 Theory

### 3.1 Energy density

The Gibbs free energy of mixing (Δ_*mix*_*G*) is defined as the potential energy that is released after the spontaneous mixing of two solutions of salt with differing concentrations:

(1) $$\Delta_{mix}G\equiv \Delta G_{B}-\left(\Delta G_{C}+\Delta G_{D}\right)$$

where the subscripts C and D relate to the concentrate and diluate and B refers to the final mixed solution. If the solutions are considered to be ideal there is no enthalpy of mixing (Δ*H* = 0) and the Gibbs free energy of mixing can therefore be calculated from the molar entropy of each solution as [39]:

(2) $$\Delta_{mix}G=-\left(n_{C}+n_{D}\right)T\Delta S_{B}-\left(-n_{C}T\Delta S_{C}-n_{D}T\Delta S_{D}\right)$$

where $n_{C}$ and $n_{D}$ are the total moles in the concentrate and diluate respectively (mol), *T* the temperature (K) and Δ*S* the molar entropy of each solution (J K^−1^ mol^−1^). The molar entropy is calculated as [39]:

(3) $$\Delta S=-R\underset{i}{\sum }x_{i}\;\ln x_{i}$$

where *R* is the universal gas constant (8.314 J K^−1^ mol^−1^) and *x*_*i*_ the mole fraction of each component within the solution (e.g. H_2_O, Na^+^, Cl^−^). Due to the very large number of ions and non-charged solutes within urine, and the infinitely variable concentration of these within real samples the calculation of molar entropy was simplified. The conductivity of solutions of synthetic or real urine were taken and a relative concentration of NaCl determined from a calibration curve. The entropy term was then calculated from this equivalent concentration of NaCl. The effect inclusion of multiple ionic species into this term would ultimately depend on their individual concentrations and activity coefficients. Replacing divalent ions (such as MgSO_4_) for NaCl would decrease the overall contribution to energy generation due to the relatively low activity coefficients of these ions, however, other monovalent ions such as acetate^−^ or K^+^ would have minimal effect due to possessing similar activity coefficients to Na^+^ and Cl^−^.

For the experiments conducted at a constant current in a recycle configuration, the obtained experimental energy density (J kg^−1^) of the system can be determined from [36]:

(4) $$ED=\frac{\int_{0}^{t}EIdt}{\left(m_{C}+m_{D}\right)}$$

where *E* is the potential (V), *I* the current (A), *t* the time (s) and *m* the starting mass of either the concentrate or diluate (kg). From this and the Gibbs free energy of mixing calculated using Equation (1), energy recovery can be calculated [36]:

(5) $$Energy\;recovery\;\left(\%\right)=\left(\frac{ED}{\Delta_{mix}G}\right)\times 100\;\%$$

### 3.2 Power density

For RED conducted in a single-pass configuration where the influent concentrations to the stack are continuous and therefore the available power output constant, the power density of the membrane stack (*PD*_*Stack*_, W m^−2^) has been calculated as [33,40]:

(6) $$PD_{Stack}=\frac{U_{Stack}I_{Stack}}{2NA}=\frac{U_{stack}I_{d}}{2N}$$

where *U*_*Stack*_ is the voltage (V) over the membrane stack, *I*_*Stack*_ is the current (A) scanned, *A* is the cross sectional area of one membrane (m^2^), *N* is the number of cell pairs in the stack and *I*_*d*_ the current density representing the current normalised to membrane area (A m^−2^).

For a system where the influent concentrations will be continuously changing such as the experiments conducted in a recycle configuration with feedwaters recirculating through the RED stack, the voltage will be constantly changing due to a continuous change in solution ionic concentration, as such there will be a continual change in power. Therefore, the average power density can be used over the discharge of the salinity gradient of the finite volumes of solution [36]. The average power density (*PD*_*avg*_, W m^−2^) has been calculated as [36]:

(7) $$PD_{avg}=\frac{1}{t}\overset{t}{\underset{0}{\int }}\frac{U_{Stack}I_{Stack}}{2NA}dt$$

where *t* is the time taken for the discharge. The energy extraction efficiency (*η*) is determined by the ratio of the electric power harvested by the RED stack over the potential Gibbs power (P_G_) released [34]:

(8) $$\eta =\frac{P_{Stack}}{P_{G}}$$

The theoretical Gibbs free energy that is released per second within the RED cell from the solutions can be calculated by [34,35]:

(9) $$P_{G}=J_{w}\left(-\Delta \mu_{w}\right)+J_{s}\left(-\Delta \mu_{s}\right)$$

where *J*_*w*_ is the total water flux (mol m^−2^ s^−1^), Δ*μ*_*w*_ difference in chemical potential of water, *J*_*s*_ is the total salt flux (mol m^−2^ s^−1^) and Δ*μ*_*s*_ the difference in chemical potential of the salt. The total water flux can be calculated from the following [35,41]:

(10) $$J_{w}=2L_{p}\left(-\Delta \mu_{w}\right)+J_{s}t_{w}M$$

where *L*_*p*_ is the average water permeability coefficient of both the anion and cation exchange membranes (kg m^−2^ s^−1^ kg ^−1^), *t*_*w*_ is the number of water molecules transported with salt ions across the membrane (mol_water_ mol_salt_^−1^) and M is the molar mass of water (kg mol^−1^). The difference in chemical potential of water of the two solutions is calculated with [35,41]:

(11) $$\Delta \mu_{w}=-vRT\left(\emptyset_{C}C_{C}-\emptyset_{D}C_{D}\right)$$

where ∅ is an osmotic coefficient. The total salt flux can be calculated using [35,41]:

(12) $$J_{s}=\frac{I_{d}}{F}+2\frac{P_{s}}{\delta_{m}}\left(C_{C}-C_{D}\right)$$

where *I*_*d*_ is the current density (A m^−2^), F is the Faraday constant (96485.33C mol^−1^), P_s_ is the average salt permeability coefficient for the anion and cation exchange membranes (m^2^ s^−1^), *δ*_*m*_ is the average membrane thickness of the anion and cation exchange membranes (m). The chemical potential difference of salt in two solutions that are separated by a membrane has been calculated using [35,41]:

(13) $$\Delta \mu_{s}=vRT\;\ln\left(\frac{\gamma_{C}C_{C}}{\gamma_{D}C_{D}}\right)$$

where *v* is the number of moles of ions in 1 mol of salt, *R* is the ideal gas constant (8.314 J K^−1^ mol^−1^), T is the temperature (K), C is the concentration of the concentrate and diluate denoted C and D respectively (mol L^−1^) and *γ* an activity coefficient to account for the non-ideal behaviour of the solutions. Activity coefficients have been estimated for NaCl solutions using the Pitzer model (Section S4) [[34], [35], [36],^42^,^43^].

### 3.3 Open circuit voltage

For RED the open circuit voltage (*OCV*, V) is the electrochemical potential difference across the stack. Assuming ideal solutions of differing concentrations of a single salt either side of a perfectly selective membrane the *OCV*_*i*_ across that membrane can be calculated from the Nernst equation [24]:

(14) $$OCV_{i}=\frac{RT}{zF}\ln\frac{\gamma_{C}C_{C}}{\gamma_{D}C_{D}}$$

where *z* is the valency of the ion (e.g. Na^+^ = +1), *F* is the Faraday constant (96485C mol^−1^), γ is the mean ionic activity coefficient of the counter-ion (the ion with opposite charge to the membrane, dimensionless) and *C* the concentration of the counter-ion in either the concentrate or diluate (mol L^−1^). To calculate the potential across a RED stack this calculated *OCV*_*i*_ can be multiplied by the number of membranes in the stack, therefore [24]:

(15) $$OCV_{ideal}=2N\cdot OCV_{i}$$

Calculation of *OCV*_*i*_; however, becomes increasingly onerous when considering complex waters consisting of many ions as each counter-ion has a unique concentration gradient across the membrane. Multivalent ions have been shown to decrease the stack voltage and therefore power density [[44], [45], [46]]. Counter-ions are exchanged across the membrane until an equilibrium in chemical potential is achieved where each ionic species has an equal OCV and therefore the uphill transport of divalent ions exchanging for a number of monovalent ions can occur [45,47,48]. Kingsbury et al. have reported the calculation of *OCV*_*i*_ when a greater number of counter-ions are present [24]. through estimating the *OCV*_*ideal*_ from the conductivity (κ) of the concentrate and diluate [24]:

(16) $$OCV_{cond}=2N\frac{RT}{zF}\ln\frac{\kappa_{C}}{\kappa_{D}}$$

The permselectivity (α, %) of an ion exchange membrane represents its ability to reject co-ions compared to that of an ideal selective ion exchange membrane (which will completely reject co-ions). Defined as the ratio of the measured and calculated ideal OCV it has therefore been calculated using: [24]:

(17) $$\alpha =\left(\frac{OCV_{exp}}{OCV_{cond}}\right)\times 100\;\%$$

where *OCV*_*exp*_ is the experimentally determined open circuit voltage (V), *OCV*_*cond*_ the open circuit voltage calculated from the conductivity of the concentrate and diluate, and the permselectivity (α, %) characterises the average over all the cation and anion exchange membranes within the stack.

## 4 Results and discussion

### 4.1 Power density from urine approaches sodium chloride

**Figure 3:**
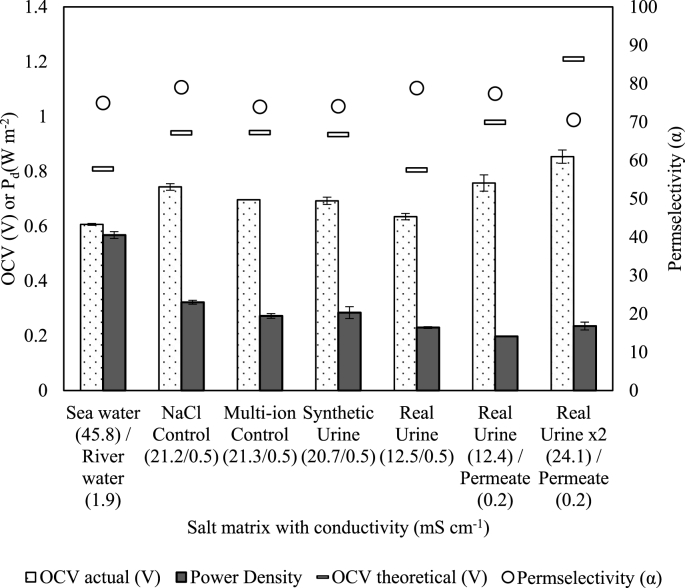
Influence of urine multivalent ions and organics (Table 1) on open circuit voltage (OCV), permselectivity and power density (P_d_). Single pass mode.

An initial benchmarking experiment using sea water/river water (45.8 and 1.9 mS cm^−1^ respectively) was carried out, achieving a power density of 0.57 W m^−2^, which is comparable to the literature using the same membranes (Table S2). The NaCl control, which was characterised by a conductivity around that of human urine but comprising only NaCl (20.7 mS cm^−1^), achieved a power density of 0.32 W m^−2^, which can be expected since the conductivity is less than half that of sea water. A multivalent ion control was subsequently evaluated, which comprised comparable conductivity (21.3 mS cm^−1^) but with a lower NaCl fraction, and the inclusion of divalent salts (Table 1). This introduced a small immediate reduction in OCV, permselectivity and power density (Fig. 3). Multivalent ions have been shown to decrease the stack voltage and therefore power density, when transported from the diluate to the concentrate due to uphill transport [[44], [45], [46]]. However, comparable OCV, permselectivity and power density was achieved with synthetic urine (20.7 mS cm^−1^), despite comprising a more complex salt matrix, which included organic salts and organic compounds (Table 1); where in contrast, a considerable organic concentration has been previously associated with fouling [24,49]. No significant change in performance was observed between experiments when the various inorganic and organic constituents of urine were included within the salt matrix, as post use characterisation of the membrane stack with pure NaCl solutions (Seawater/River Water) to determine loss in performance from membrane fouling resulted in the same performance as before use (OCV: ± 0.02 V, P_d_: ± 0.01 W). This indicated an absence of significant fouling within the relatively short experimental timeframes; however, longer term studies will be required to reveal the extent to which this “absence of fouling” would last, particularly at higher solute concentrations. Electrodialysis studies have shown that organic fouling behaviour is determined by specific properties of organic matter and not necessarily concentration [[50], [51], [52]]. Kingsbury et al. suggested there is a negative linear relationship between permselectivity and UV_254nm_ absorbing organics within the diluate but little relationship between their concentration in the concentrate and permselectivity [24]. Therefore, it is proposed that the limited organic concentration in the diluate limited any detrimental impact on the attainable OCV. When comparing synthetic urine to real urine using the same diluate solution comprising 0.004 M NaCl (0.5 mS cm^−1^), the power density was 20% greater for the synthetic, which we propose is due to a higher concentrate conductivity (21 and 12 mS cm^−1^ respectively). However, the conductivity was 40% higher, which should have resulted in a proportionately lower power density, which we ascribe to the wider transient properties of urine [17]. Real urine was subsequently evaluated using MD permeate as the diluate (0.2 mS cm^−1^), which resulted in a 13% reduction in power density (Fig. S3), most likely attributable to the increased solution resistance in the diluate compartment due to a lower conductivity. Importantly, the power densities achieved using urine were not markedly below those nominally observed for NaCl at an equivalent conductivity suggesting that whilst important, the complexity of the feed matrix is not the primary factor in determining the OCV.

**Fig. 4:**
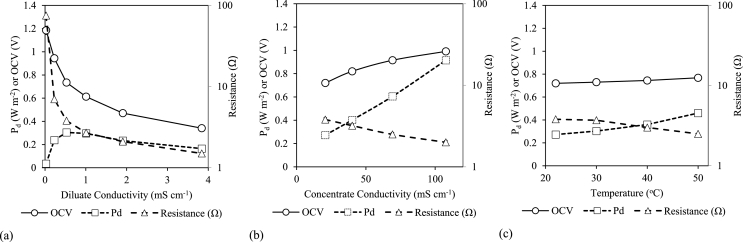
Influence of (a) diluate concentration; (b) synthetic urine concentration and (c) temperature on power density (P_d_), open circuit voltage (OCV) and resistance. Single pass mode. Diluate 0.5 mS cm^−1^, concentrate 21 mS cm^−1^ and temperature 22 °C, unless stated otherwise.

The impact of solution concentration was further investigated in order to understand the dynamic conditions within MD-RED associated with gradual ion transfer into the permeate in recycle mode (Fig. 4a), and MD retentate concentration factors (Fig. 4b). Although comprising the greatest concentration gradient and therefore highest electromotive force, the initial diluate conductivity (0.026 mS cm^−1^) demonstrated a detrimental effect on power density (0.025 W m^−2^) due to the large internal resistance (75 Ω), attributable to high resistance of the diluate. Power density peaked (0.3 W m^−2^) at a diluate conductivity of 0.5 mS cm^−1^ and declined to 0.165 W m^−2^ as the rapid decline of internal resistance to 1.5 Ω at 3.8 mS cm^−1^ is offset by the decrease in electromotive force [53]. Weiner et al. reported an optimal diluate concentration of 0.01 M NaCl [21,54], whilst Veerman et al. considered 0.005 M NaCl to be optimum, both studies using 0.5 M NaCl (seawater) as the high concentration [21,55]. Whilst the optimum conductivity range for urine is greater than the conductivity of the real MD permeate, this concentration can be approached as solution mixing progresses during recycling. When increasing the concentrate conductivity by a factor of eight, power density responded linearly by a factor of 3.4, which corresponded with reduced internal resistance (52%) and OCV increased (38%) as a result of a higher concentration gradient (Fig. 4b). The slightly disproportionate increase in OCV illustrates reduced membrane permselectivity at higher retentate concentrations, associated with non-ideal salt transport [21], potentially attributed to swelling of the ion exchange membranes. In addition increased organic concentration within the HC (such as urea) will have a detrimental impact through increasing the entropy of the solution and therefore increasing water transport and/or membrane fouling whilst not contributing to energy generation. Zhu et al. reported that 3.6 M NaCl was the upper concentrate boundary condition for RED stack power production, theoretically allowing for the urine to be concentrated by a factor of 18 (∼0.2 M starting concentration) through MD before dramatically affecting power density [40], which demonstrates that RED can undergo substantial optimisation for power density and energy recovery in a MD-RED hybrid configuration.

Temperature increased power density and reduced overall cell resistance (Fig. 4c). Increased temperature facilitates ion mobility which reduces ion transport resistance through the membrane, ohmic resistance and hydrodynamic losses (from reduced viscosity) [21,29]. From 22 °C to 50 °C, resistance decreased by 66% which coincided with a power density increase of 70%. The direct relationship between resistance and power density has also been observed by Tedesco et al., who reported an internal resistance decline of 30–50% with increased power density of 40–50% when increasing temperature from 20 °C to 40 °C, using brackish water and brine [56]. Benneker et al. demonstrated a 38% increase in power density from 20 °C to 40 °C (sea water/river water) compared to a 32% increase from 22 °C to 40 °C in this work, demonstrating relatability to other salt matrices [57]. Open circuit voltage was minimally affected within this temperature range (22–50 °C) indicating that permselectivity was not compromised. Daniilidis et al. reported that energy efficiency and permselectivity were severely affected above 50 °C, due to ionic shortcuts and therefore 50 °C was a suitable boundary condition for MD-RED [58]. Such a temperature is accessible by waste heat and provides the opportunity to increase power output and accelerate energy recovery.

### 4.2 Hydrodynamic optimisation is critical for energy recovery

**Figure 5:**
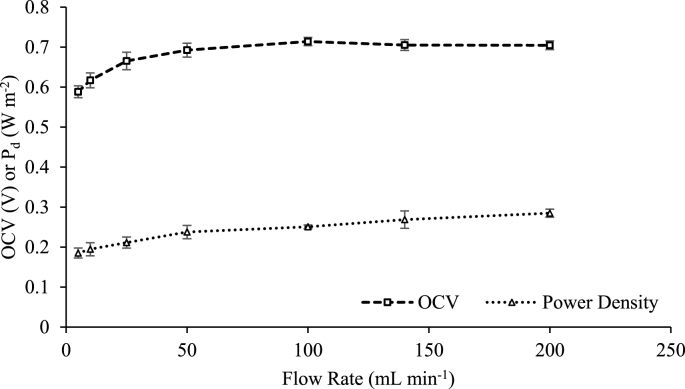
Effect of varying feed flowrates on open circuit voltage (OCV) and power density (P_d_). Synthetic urine concentrate, 0.004 M NaCl diluate, single pass mode.

**Fig. 6:**
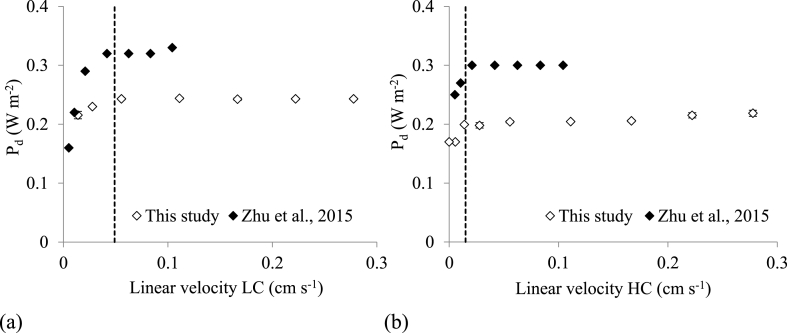
Power density as a function of linear velocity (a) when LC flowrate is variable and HC flowrate is maintained at 50 mL min^−1^ and (b) when HC flowrate is variable and LC flowrate is maintained at 50 mL min^−1^. Synthetic urine concentrate, 0.004 M NaCl diluate, single pass mode.

Hydrodynamic conditions were trialled in single pass and recycle mode to understand the impact on power density and energy recovery. In single pass mode, power density and OCV increased by 54% and 18% respectively from operation between 5 and 200 mL min^−1^ (Fig. 5), and plateaued at 50 mL min^−1^. Increasing solution flowrate improved hydrodynamic mixing, subsequently reducing concentration polarisation and boundary layer thickness, therefore maintaining the maximum concentration gradient [21,59]. As hydrodynamic and pumping losses occur at higher flowrates, there is a compromise for net power density. As a plateau was approached for power density with flowrates greater than 50 mL min^−1^, the cell was subsequently trialled at 50 mL min^−1^. Zhu et al. identified that pumping losses were reduced further by operating the diluate solution at a higher linear velocity than the concentrate solution [33]. Results were comparable to Zhu et al. and demonstrated similar linear velocity boundary conditions (represented as the dotted lines on Fig. 6) at 0.4 cm s^−1^ LC and 0.015 cm s^−1^ HC which equates to 10 mL min^−1^ LC and 2.5 mL min^−1^ HC according to channel thicknesses of 0.3 mm [33]. Subsequently, the power densities for operating at 10 mL min^−1^ HC and 2.5 mL min^−1^ were comparable at 0.23 W m^−2^ (Fig. S4). The advantage of the higher diluate linear velocity, can be ascribed to the reduction in fluid resistance introduced through reducing the concentration gradient in the boundary layer that develops in the diluate channel. A reduction in concentrate channel flowrate can therefore reduce pumping energy.

### 4.3 Maximising energy recovery from a finite volume in recycle mode

**Fig. 7:**
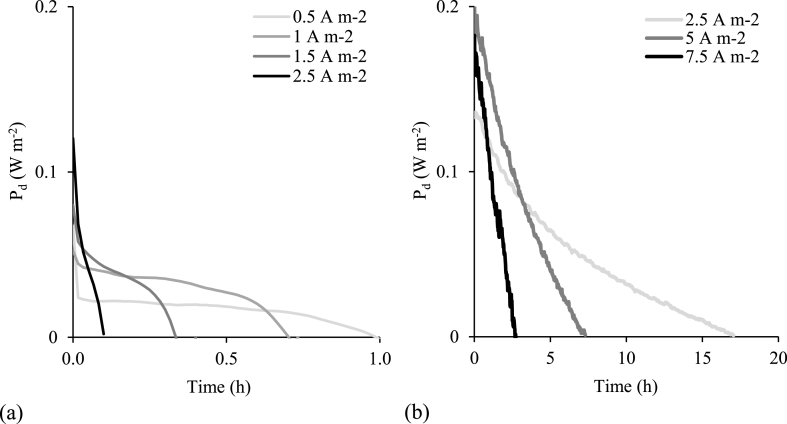
Comparison of (a) low and (b) high flowrates in recycle mode until power fully extracted, at varying current draws (A m^−2^). High flowrates (concentrate and diluate at 140 mL min^−1^), low flow rates (2.5 mL min^−1^ concentrate/10 mL min^−1^ diluate). Synthetic urine concentrate, 0.004 M NaCl diluate.

**Figure 8:**
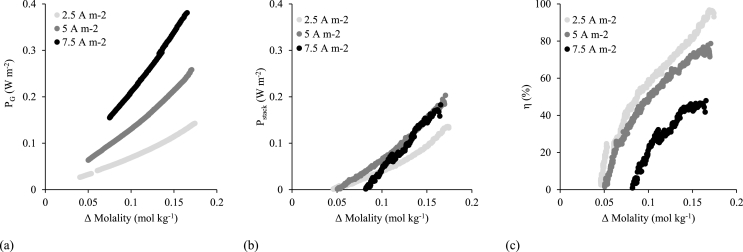
(a) Theoretical Gibbs power, (b) experimentally obtained power and (c) energy extraction efficiency (η) as a function of the molality difference between HC and LC at varying current densities (2.5 A m^−2^, 5 A m^−2^ and 7.5 A m^−2^). Concentrate and diluate flowrate is 140 mL min^−1^. Synthetic urine concentrate, 0.004 M NaCl diluate, recycle mode.

Within ISO standard 30500 on ‘non-sewered sanitation’, a single use system is engineered to withstand an average of ten users per day, which based on the median daily production of human urine of 1.4 L cap^−1^ day^−1^, provides a total volume of around 15 L d^−1^ required for treatment [60]. This is in contrast to the traditional electrolytic solutions ordinarily associated for energy generation within RED such as sea water and river water as these have effectively infinite available volumes. Due to the limited volume, and to elicit maximum energy recovery from the available MD urine concentrate, a closed loop configuration is proposed (recycle mode). The system can therefore be described similar to a battery with the state of charge (SOC) described as the difference in concentration between the concentrate and diluate. In recycle mode, a contrasting outcome was evidenced between high and low flowrates (Fig. 7). Due to the evolving concentration gradient and increasing LC ohmic resistance [33], low flowrates (2.5 mL min^−1^ concentrate/10 mL min^−1^ diluate) provided an average power density of 0.043 W m^−2^ for 0.1 h, compared to 0.048 W m^−2^ for 17 h (140 mL min^−1^ both compartments). Therefore, higher flowrates were required to accommodate for the dynamic conditions experienced in recycle mode, particularly the increasing diluate concentration. According to the theoretical values calculated from the Gibbs power, higher current densities achieve greater power densities as the concentrate and diluate approach equilibrium (Fig. 8a). When operating current draws of 7.5, 5.0 and 2.5 A m^−2^, initial power densities (at 0.175 Δ mol kg^−1^) were 0.38, 0.26 and 0.14 W m^−2^ respectively. For comparison, respective experimentally obtained power densities were 0.18, 0.20 and 0.13 W m^−2 (^Fig. 8b) and reached full extraction at 0.08, 0.05 and 0.05 mol kg^−1^ (Δ molality). The energy extraction efficiency (η) illustrates how closely experimentally obtained power represents the theoretically available power at varying current draws (Fig. 8c). In this study, the lowest current density (2.5 A m^−2^) provided the overall greatest η, particularly until reaching a molality difference of 0.1 mol kg^−1^ where η remained above 60%.

In recycle mode, total power dissipation is affected by osmotic transport, non-ideal salt transport and internal resistance. Osmotic transport was insignificant, as the maximum concentration demonstrated was 0.2 M, providing an osmotic pressure of ∼9.9 bar. Previous literature has reported that higher concentrate concentrations facilitate water transport, particularly higher than 1.5 M (urine concentration factor of 7.5; ∼70 bar osmotic pressure) [35,36], where non-ideal salt transport and internal resistance provide little contribution. Egmond et al. [34] demonstrated that increased temperature also exaggerates the rate of water transport, predominantly at lower current draws which require a greater recycle time to achieve full discharge. Another study by Egmond et al. [35] identified that non-ideal salt transport energy dissipation is also linked to higher concentration gradients, due to the facilitation of co-ion diffusion. As osmotic transport and non-ideal salt transport did not play a role in the reduction of η in this study, dissipation was primarily caused by internal resistance which was prevalent at higher current draws following Ohm's law (Fig. 8c). Therefore, when considering operation at higher temperatures or with greater retentate concentration factors required to maximise power output, higher current draws should be trialled, which can increase the rate of electro-osmosis to negate the rate of water transport, whilst identifying a compromise with internal resistance at higher current draws [35].

**Fig. 9:**
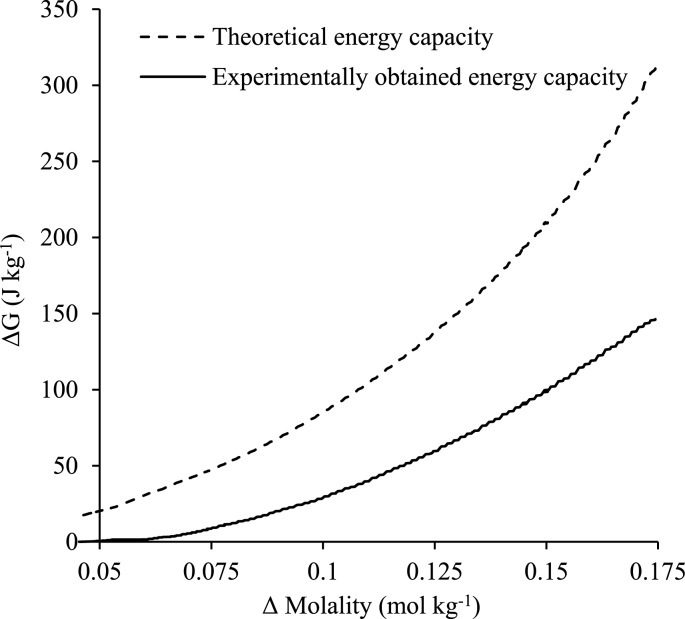
Comparison of theoretical and experimentally obtained energy densities at a starting molality difference of 0.175 mol kg^−1^ and operating 2.5 A m^−2^ current draw. Synthetic urine concentrate, 0.004 M NaCl diluate, recycle mode.

Fig. 9 illustrates the energy recovered (47%) using the most efficient energy extraction current draw (2.5 A m^−2^) against the theoretical Gibbs free energy, under the conditions trialled (Δm = 0.175 mol kg^−1^, 22 °C), which is consistent with other similar studies obtaining between 45 and 60% when equal volumes of concentrate and diluate are utilised [36]. Vermaas et al. suggest that higher energy recoveries can be obtained when the diluate volume is relatively larger than the concentrate volume if osmotic transport occurs [61]. For urine MD-RED, this can be straightforwardly achieved by greater retentate concentration factors to increase permeate volume.

## 5 Conclusions

This study has provided the first successful demonstration of hybrid MD-RED using urine, to create a synergistic relationship in which high quality water can be produced using waste heat, and the subsequent salinity gradient generated in membrane distillation utilised for the production of electrical energy. The urine salt matrix combined with the high quality MD permeate (COD 253 mg L^−1^, conductivity 0.21 mS cm^−1^) provided comparable power densities (0.2 W m^−2^) to a simple NaCl matrix (0.32 W m^−2^) despite the complexity of the salt matrix, organic salts and organic constituents. Around 47% of the Gibbs free energy available was recoverable, which can be used in low power fluidic devices to permit overall water recovery from MD. For example, the mixing energy from l L urine and 1 L permeate is sufficient to operate an axial fan for sweep gas, or a micro-pump for the provision of liquid transport at head pressures and flow rates up to 500 mbar and 350 mL min^−1^ respectively. An increase in system surface area and cell pair number will scale the device to increase both current densities and voltage achievable to power larger scale devices [62]. Concentrating the retentate can improve power density, as can operating the feedside of RED at temperatures comparable to those employed in MD, which evidences system compatibility. Whilst not observed within the boundary conditions evaluated within the present study, limiting phenomena such as non-ideal salt and water transport [[34], [35], [36]] will define the upper limit to operation without identifying membrane materials which possess preferential permselectivity, resistance and water permeability criteria. However, discharging energy over shorter timescales will minimise parasitic and hydrodynamic losses associated with sustained operation, but would favour peak power in short cycles rather than sustained energy delivery which has implications for process operation and energy storage. Up to 80% of the available energy is extractable when the concentration difference between the two solutions is halfway towards equilibrium [34] which implies that energy recovery can be optimised with limited effect on permeate quality. While further optimisation, including technology scale-up and long term field trials are warranted, the partnership between MD and RED has the potential to provide water for safe discharge or reuse within small scale decentralised sanitation systems, using waste heat as the primary energy source, whilst providing sufficient electrical energy to support the limited power requirements for off-grid operation, thus overcoming the present technical constraints of the low income country setting.
