# Supplementary material for: Hybrid membrane distillation reverse electrodialysis configuration for water and energy recovery from human urine: An opportunity for off-grid decentralised sanitation
Source: J Memb Sci. 2019 Aug 15;584:343–52. doi: 10.1016/j.memsci.2019.05.010 (PMC6558964; doi:10.1016/j.memsci.2019.05.010)
Supplement: Multimedia component 1 [file mmc1.docx]

**Supplementary Material**

# Hybrid membrane distillation reverse electrodialysis configuration for energy recovery and concentrate management from human urine in decentralised treatment technologies

E. Mercer^a^, C.J. Davey^a^, D. Azzini^b^, A.L. Eusebi^b^, R. Tierney^a^, L. Williams^a^, A. Kolios^c^, A. Parker^a^, S. Tyrrel^a^, E. Cartmell^d^, M. Pidou^a^, E.J. McAdam^a,^*

^a^Cranfield Water Science Institute, Vincent Building, Cranfield University, Bedfordshire, UK

^b^Department of Materials, Environmental Sciences and Urban Planning, Università Politecnica delle Marche, Piazza Roma, Ancona, Italy

^c^Naval Architecture, Ocean and Marine Engineering, University of Strathclyde, Glasgow, UK

^d^Scottish Water, Castle House, Carnegie Campus, Dunfermline, UK

*Corresponding author e-mail: [*e.mcadam@cranfield.ac.uk*](mailto:e.mcadam@cranfield.ac.uk)

**Section S1. Membrane distillation**

|  |
| --- |

Figure S1. Schematic of the vacuum membrane distillation setup used to concentrate real urine in this study.

**Section S2. Polarisation curves representing the influence of different solution chemistries**

Figure S2. Polarisation curves obtained at (a) varying synthetic urine concentration, (b) varying diluate concentration and (c) varying solution temperature.

| (a) | (b) |
| --- | --- |

Figure S3. Real urine (1x and 2x concentrated) and membrane distillation (MD) permeate polarisation curves (b) visual comparison of real urine and membrane distillation permeate solutions. Real urine concentration was 12.45 mS cm^-1^, 2x concentrate was 24.1 mS cm^-1^, and permeate was ~0.2 mS cm^-1^.

**Section S3. Polarisation curves comparing hydrodynamic conditions**

|  |
| --- |

Figure S4. Single pass polarisation curves comparing HC (high concentration) and LC (low concentration) flowrate regimes: HC and LC compartments both at 50 mL min^-1^ and HC at 2.5 mL min^-1^ with LC at 10 mL min^-1^.

| **Section S4. Characteristics and reported performance of the commercially available membranes used in this study**  Table S1. Properties of the commercial ion exchange membranes used in this study. | | | | | |
| --- | --- | --- | --- | --- | --- |
| Membrane Type | Model | Company | Thickness (µm) | Resistance (Ω.cm^2^) | Permselectivity (%) |
| Anionic | AMX | Neosepta | 114^1^ | 2.4^1^ | 95^2^ |
| Cationic | CMX | Neosepta | 117^1^ | 3^1^ | 97^2^ |

0.5 M NaCl at 25 °C;^1^ 0.1 / 0.01 M NaCl at 25 °C.^2^

| Table S2. Reported power densities for Neosepta AMX/CMX ion exchange membranes. | | | | | | |
| --- | --- | --- | --- | --- | --- | --- |
| Effective membrane area (cm^2^) | Spacer thickness (µm) | Number of cells | Salt matrix  (LC / HC  g L^‑1^ NaCl) | Power density (W m^-2^) | Flow velocities (cm s^-1^) | Reference |
| 100 | 300 | 5 | 1 / 30 | 0.56 | 0.7 | This study |
| 100 | 200 | 5 | 1 / 30 | 1.07 | 0.27–1.7 | Guler *et al*.^3^ |
| 100 | 320 | 3 | 1 / 30 | 0.8 | 0.06-0.83 | - [Długołęcki](http://www.sciencedirect.com/science/article/pii/S0376738809007418) *et al*.^4^ |
| 100 | 320 | 3 | 1 / 30 | 0.27 | 0.06-0.83 | - [Długołęcki](http://www.sciencedirect.com/science/article/pii/S0376738809007418) *et al*.^4^ |
| 100 | 200 | 5 | 1 / 30 | 0.65 | 1.17 | Veerman *et al*.^5^ |

**Section S5. The Pitzer Model**

The Pitzer model was utilised for calculating the activity coefficient and osmotic coefficient of electrolyte solutions. For the multicomponent electrolyte solutions of synthetic urine and urine a solution of NaCl in water of equivalent conductivity was assumed. The calculation of activity and osmotic coefficients of multicomponent electrolyte solutions has been described elsewhere but the approach was not adopted for this work. The calculations utilised are outlined below and further details on the Pitzer model can be found in ^6,7^.

| $\emptyset-1=\left\vert z_{m}z_{x} \right\vert f^{\emptyset}+m\left( \frac{2v_{M}v_{X}}{v} \right)B_{MX}^{\emptyset}+m^{2}\frac{2\left( v_{M}v_{X} \right)^{\frac{3}{2}}}{v}C_{MX}^{\emptyset}$ | Equation S1a. |
| --- | --- |

| $\ln\gamma=\left\vert z_{m}z_{x} \right\vert f^{\gamma}+m\left( \frac{2v_{M}v_{X}}{v} \right)B_{MX}^{\gamma}+m^{2}\frac{2{(v_{M}v_{X})}^{\frac{3}{2}}}{v}C_{MX}^{\gamma}$ | Equation S1b. |
| --- | --- |

| $v= v_{m}+ v_{x}$ | Equation S1c. |
| --- | --- |

| $f^{\gamma}=-A^{\phi}[\frac{I^{\frac{1}{2}}}{1+{bI}^{\frac{1}{2}}}+\frac{2}{b}ln(1+{bI}^{\frac{1}{2}}$)] | Equation S1d. |
| --- | --- |

| $f^{\emptyset}= -A^{\emptyset}\frac{I^{\frac{1}{2}}}{1+{bI}^{\frac{1}{2}}}$ | Equation S1e. |
| --- | --- |

| $B_{MX}^{\gamma}={2\beta}_{MX}^{0}+ \frac{{2\beta}_{MX}^{1}}{\alpha^{2}I}\left[ 1-e^{{\alpha I}^{\frac{1}{2}}}\left( 1+{\alpha I}^{\frac{1}{2}}-\frac{1}{2}\alpha^{2}I \right) \right]$ | Equation S1f. |
| --- | --- |

| $B_{MX}^{\emptyset}= B_{MX}^{0}+ {B_{MX}^{1}e}^{-\alpha I^{\frac{1}{2}}}$ | Equation S1g. |
| --- | --- |

| $C_{MX}^{\gamma}= \frac{3}{2}C_{MX}^{\emptyset}$ | Equation S1h. |
| --- | --- |

| $I= \frac{1}{2}\Sigma m_{i}z_{1}^{2}$ | Equation S1i. |
| --- | --- |

Where:

$A^{\emptyset}$ = 0.392 for water at 25°C

$b$ = 1.2

$\alpha$ = 2

$B_{MX}^{0}$ = 0.0765, $B_{MX}^{1}$ = 0.2664, $C_{MX}^{\gamma}$ = 0.00127, $m$ = 6 are Pitzer parameters for NaCl.

$v_{M}$ and $v_{X}$represent number of ions in the salt formula with respective charges as $z_{m}$ and $z_{x}$.

**References**

1 Astom Corporation, 2013, 40.

2 R. K. Nagarale, G. S. Gohil and V. K. Shahi, *Adv. Colloid Interface Sci.*, 2006, **119**, 97–130.

3 E. Güler, R. Elizen, D. A. Vermaas, M. Saakes and K. Nijmeijer, *J. Memb. Sci.*, 2013, **446**, 266–276.

4 P. Długołęcki, J. Dąbrowska, K. Nijmeijer and M. Wessling, *J. Memb. Sci.*, 2010, **347**, 101–107.

5 J. Veerman, R. M. de Jong, M. Saakes, S. J. Metz and G. J. Harmsen, *J. Memb. Sci.*, 2009, **343**, 7–15.

6 K. S. Pitzer and G. Mayorga, *J. Phys. Chem.*, 1973, **77**, 2300–2308.

7 W. J. van Egmond, M. Saakes, S. Porada, T. Meuwissen, C. J. N. Buisman and H. V. M. Hamelers, *J. Power Sources*, 2016, **325**, 129–139.
